# Rethink poverty during age: Time for Umberto D. again

**DOI:** 10.1016/j.tjfa.2026.100197

**Published:** 2026-09-04

**Authors:** Emanuele Marzetti, Hélio Jose Coelho-Junior

**Affiliations:** a Fondazione Policlinico Universitario A. Gemelli IRCCS, Rome, Italy; b Department of Geriatrics, Orthopedics and Rheumatology, Università Cattolica del Sacro Cuore, Rome, Italy

## Dear editor

Poverty among older adults is frequently accompanied by social isolation, loneliness, inadequate social support, and loss of dignity, dimensions that are now recognized as core components of social frailty [1,2]. According to the United Nations [3], the risk of poverty increases with age, with the oldest-old (≥80 years) being particularly vulnerable due to reduced capacity to work, depletion of savings, and increasing need for health and long-term care services. This demographic group is also the fastest growing segment of the older population worldwide, with projections indicating a marked increase in its size in the coming decades. Although these challenges may appear to be modern concerns [4], they were vividly portrayed more than seventy years ago in *Umberto D.* (1952), one of the masterpieces of Italian neorealism.

The film tells the story of Umberto Domenico Ferrari, a retired civil servant who, after more than 30 years of work, struggles to survive on an inadequate pension. He lives in a rented room with his dog, Flike, his only true companion. At the beginning of the film, Umberto joins a protest of pensioners demanding better retirement benefits. His financial situation is precarious. He cannot afford the rent, has difficulties paying his bills, and often lacks enough money for food. Despite his own hardship, he continues to share what little he has with his dog.

Things gradually become worse. His landlady increases the rent and repeatedly pressures him to pay his debts. He then falls ill and is admitted to hospital. Even there, he encounters indifference. His suffering is minimized, almost as if illness and discomfort are expected consequences of old age.

What makes *Umberto D.* so powerful is not only poverty itself but the loneliness that accompanies it. Few people seem genuinely interested in Umberto's situation. His former colleagues are unable or unwilling to help. His landlady shows no compassion. Upon returning from the hospital, he discovers that his dog has disappeared and that his room is being altered without his consent, reinforcing the feeling that he no longer has a place in the world.

To make ends meet, Umberto sells his belongings, including personal items that represent much of his past. Eventually, driven by desperation, he considers begging in the street, something that deeply hurts his sense of dignity. Throughout the film, however, his main concern remains the fate of Flike. Even when his own future appears hopeless, he worries about who will care for his dog.

More than seventy years after its release, *Umberto D.* remains remarkably relevant. The film reminds us that poverty in old age is not only a matter of insufficient income. It is also about social exclusion, loneliness, loss of dignity, and the feeling of becoming invisible. All dimensions related to the concept of social frailty [1,2]. De Sica portrays with extraordinary sensitivity the vulnerability of an older man who no longer seems to have a place in society.

As populations age worldwide, *Umberto D.* invites us to reflect on how we care for older adults facing financial hardship. While the social and economic context has changed since 1952, many of the challenges portrayed in the film remain familiar today. For this reason, revisiting *Umberto D.* may help us better understand the human consequences of poverty in later life and the importance of preserving dignity and social support in old age.

## Ethical approval

No ethical approval is requested for editorial articles.

## Funding

This research did not grant any external funding.

## Declaration of generative AI and AI-assisted technologies in the manuscript preparation process

Neither artificial intelligence (AI) nor AI-assisted technologies were used in the preparation of this manuscript.

## CRediT authorship contribution statement

**Emanuele Marzetti:** Conceptualization, Data curation, Formal analysis, Investigation, Writing – original draft, Writing – review & editing. **Hélio Jose Coelho-Junior:** Conceptualization, Data curation, Formal analysis, Writing – original draft, Writing – review & editing.

## Declaration of competing interest

The authors have declared no conflict of interest.
